# Morphometric analysis of the placenta in the New World mouse *Necromys lasiurus* (Rodentia, Cricetidae): a comparison of placental development in cricetids and murids

**DOI:** 10.1186/1477-7827-11-10

**Published:** 2013-02-21

**Authors:** Phelipe O Favaron, Andrea M Mess, Moacir F de Oliveira, Anne Gabory, Maria A Miglino, Pascale Chavatte-Palmer, Anne Tarrade

**Affiliations:** 1 Department of Surgery, School of Veterinary Medicine, University of Sao Paulo, Av. Prof. Dr. Orlando Marques de Paiva, 87, Cidade Universitária, São Paulo, SP, CEP 05508-270, Brazil; 2 Department of Animal Science, Universidade Federal Rural do Semi-Árido, Mossoró, Rio Grande do Norte, 59625-900, Brazil; 3 INRA, UMR 1198 Biologie du Développement et Reproduction, Jouy-en-Josas, F-78352, France; 4 ENVA, Maisons-Alfort, F-94704, France; 5 Foundation PremUp, Paris, France

**Keywords:** Placenta, Stereology, Sigmodontinae, Decidua, Junctional zone, Labyrinth, Haemochorial placenta, Fetal vessels, Trophoblast, Evolution

## Abstract

**Background:**

Stereology is an established method to extrapolate three-dimensional quantities from two-dimensional images. It was applied to placentation in the mouse, but not yet for other rodents. Herein, we provide the first study on quantitative placental development in a sigmodontine rodent species with relatively similar gestational time. Placental structure was also compared to the mouse, in order to evaluate similarities and differences in developmental patterns at the end of gestation.

**Methods:**

Fetal and placental tissues of *Necromys lasiurus* were collected and weighed at 3 different stages of gestation (early, mid and late gestation) for placental stereology. The total and relative volumes of placenta and of its main layers were investigated. Volume fractions of labyrinth components were quantified by the One Stop method in 31 placentae collected from different individuals, using the Mercator® software. Data generated at the end of gestation from *N. lasiurus* placentae were compared to those of *Mus musculus domesticus* obtained at the same stage.

**Results:**

A significant increase in the total absolute volumes of the placenta and its main layers occurred from early to mid-gestation, followed by a reduction near term, with the labyrinth layer becoming the most prominent area. Moreover, at the end of gestation, the total volume of the mouse placenta was significantly increased compared to that of *N. lasiurus* although the proportions of the labyrinth layer and junctional zones were similar. Analysis of the volume fractions of the components in the labyrinth indicated a significant increase in fetal vessels and sinusoidal giant cells, a decrease in labyrinthine trophoblast whereas the proportion of maternal blood space remained stable in the course of gestation. On the other hand, in the mouse, volume fractions of fetal vessels and sinusoidal giant cells decreased whereas the volume fraction of labyrinthine trophoblast increased compared to *N. lasiurus* placenta.

**Conclusions:**

Placental development differed between *N. lasiurus* and *M. musculus domesticus*. In particular, the low placental efficiency in *N. lasiurus* seemed to induce morphological optimization of fetomaternal exchanges. In conclusion, despite similar structural aspects of placentation in these species, the quantitative dynamics showed important differences.

## Background

Stereology is a well-established method to extrapolate three-dimensional structural quantities from measurements on two-dimensional sectional images by using a randomized design-based approach [1,2]. It facilitates interpretations of normal and abnormal structure from a whole organ to the subcellular level [3]. Besides other organs, this approach has been used to investigate the development of placental tissues with special reference to the feto-maternal exchanges in the human placenta [3,4]. Stereology contributed to clarify key issues on normal and perturbed morphogenesis in human placentation [4-8], but this method has been also applied to placentation in animal species such as the mouse [9], the bovine, including cloned specimens [10,11], the horse [12-14] and non-human primates [15,16].

**Figure 1:**
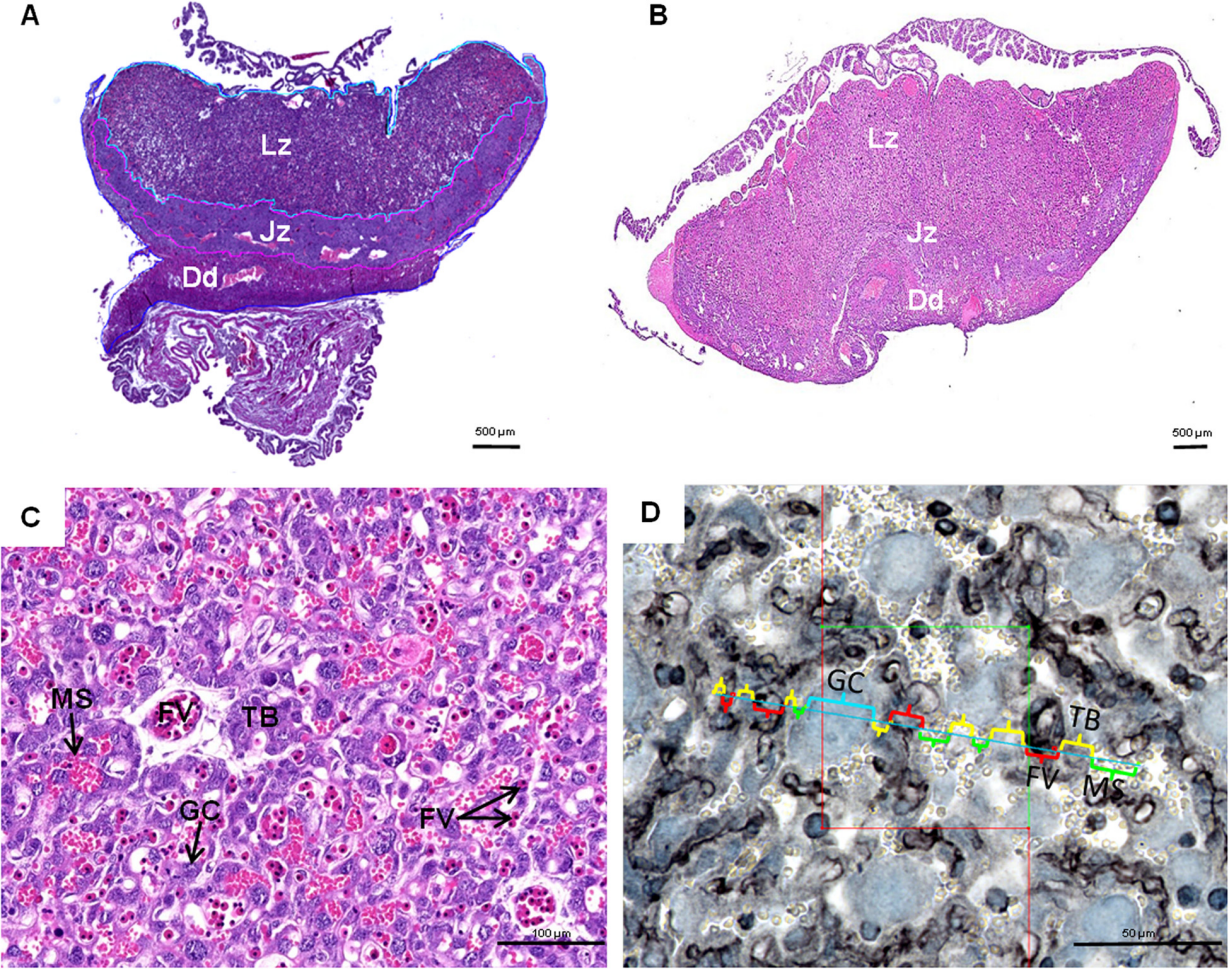
**Placental structure.** (**A**) Haematoxylin and eosin staining of *Necromys lasiurus* placenta at late gestation. The labyrinth zone, junctional zone and decidua are indicated by Lz, Jz, and Dd, respectively. (**B**) Haematoxylin and eosin staining of *Mus musculus domesticus* placenta at late gestation. (**C**) Haematoxylin and eosin staining of *N. lasiurus* labyrinth at early gestation. Labyrinthine trophoblastic layer (TB) in contact with maternal blood is indicated. Fetal vessels (FV) are distinguishable by their erythroblasts content whereas maternal space (MS) contained erythrocytes. Sinusoidal giant cells (GC) characterized by a large and dense nuclei are also mentioned. (**D**) Immunohistochemistry of vimentin on *N. lasiurus* placenta at late gestation and schematic diagram with linear dipole probes used for the One stop stereology. Positive staining is detected in the endothelium of vessel indicating the presence of fetal blood system (FV), whereas labyrinthine trophoblast borders (TB) of the maternal blood spaces (MS) and sinusoidal giant cells (GC) were vimentin-negative. In the image, the space occupied by each cell type is identified within a colorful parenthesis.

Rodents, *Mus musculus* in particular, using knock-out or targeting gene technologies, have improved our understanding on placental development and consequently on human placentation [17-20]. However, studies on quantitative aspects of normal placentation and pathological aspects are rare and refer only to the mouse [9,21-31]. Indeed, structural data on placental development are available in the mouse [18,32-37] and near relatives such as the rat [38]. Investigations in Cricetidae including New World mice or Sigmodontinae [39-44], which are close relatives of Muridae, indicated that structural aspects of placentation represented a largely conserved pattern. The chorioallantoic placenta in both groups is discoidal and organized into labyrinth, junctional zone and decidua (Figure 1A,B). Moreover, the fetomaternal interface inside the labyrinth is defined as haemochorial type (Figure 1C). We herein provide the first study on the quantitative development of main placental regions in a sigmodontine rodent of South American savannas. *Necromys lasiurus* is a small cricetid rodent commonly known as hairy-tailed akodont broadly distributed throughout South America. The average litter size is 3–6 pups and their birthweight is 2 g [45]*.* Special reference is drawn to the development of volume quantities of placental layers and components of the labyrinth as area of nutrient exchange from maternal to fetal side. As the gestational period is 23 days in *N. lasiurus*[45] and 19 days in *M. musculus domesticus*, *M. musculus* placentae collected at the end of gestation were included in our study to determine functional significances related to quantitative changes during late gestation and to find out if both taxa have similar patterns of quantitative development.

## Methods

### Animals

Altogether 31 placentae of *N. lasiurus* were obtained from a breeding group at the Universidade Federal Rural do Semi-Árido, Mossoró, Brazil. The project was approved by the Bioethic Commission of the School of Veterinary Medicine and Animal Science, University of São Paulo (Protocol number 1766/2009). The material covered three gestational phases, i.e. early gestation (10–13 days, n = 14), mid gestation (15–16 days, n = 9) and near term (21–22 days, n = 8). Gestational age was estimated by using crown-rump lengths (CRL) of fetuses in comparison to published data on the mouse [46]. Fetuses and placentae were weighed and the placental efficiency (fetal weight/placental weight) were calculated (Table 1). For comparison, four mouse placentae (*Mus musculus domesticus*, C57Bl/6 × DBA2, Harlan Laboratories, Netherlands) collected at 15.5 days of gestation (exactly date of pregnancy) at the INRA were investigated by the same methods.

**Table 1:** Fetal and placental weights (g) and feto-placental weight ratio regarded as placental efficiency in *Necromys lasiurus *(Rodentia, Cricetidae) during gestation*

	**Early gestation**			**Mid gestation**		**Near term placenta**	
	**10 days n = 4**	**11 days n = 5**	**13 days n = 5**	**15 days n = 4**	**16 days n = 5**	**21 days n = 4**	**22 days n = 4**
**Fetal weight (g)**	0,0835 ± 0,02043	0,1068 ± 0,01660	0,1028 ± 0,01307	0,5458 ± 0,01586	0,5728 ± 0,01504	0,9733 ± 0,05138	1,038 ± 0,06650
**Placental weight (g)**	0,05175 ±,00725	0,0582 ±,008766	0,0680 ±,005805	0,0900 ± 0,01235	0,1282 ± 0,02183	0,8650 ±,007382	0,9213 ± 0,02186
**Feto/Placental (g)**	0,7530 ± 0,1867	0,5807 ± 0,08380	0,7383 ± 0,1744	0,7977 ± 0,01580	0,8323 ± 0,02759	0,8947 ± 0,03743	0,9025 ± 0,07867

* Gestational age was estimated according to Evans and Sack (1973).

### Histology and immunohistochemistry

Placentae were fixed in 10% formalin, dehydrated in ethanol solutions of increasing concentration, cleared in xylene, embedded in paraplast and sectioned at 5 μm (Leica RM2155 microtome, Germany). In early stages of pregnancy, fetal erythrocytes are nucleated, thus the fetal and maternal blood systems inside the labyrinth were distinguishable using histological sections stained with haematoxylin and eosin (see Figure 1C). In mid and late gestation, the absence of erythroblasts in the fetal vessels lead us to use immunodetection of vimentin. For this, sections were incubated with the primary antibody (1:200; Clone V9, Millipore, France) overnight at 4°C. After several washed, a biotinylated secondary antibody (1:500, biotin donkey anti-mouse, Interchim, Montluçon, France) was applied for 1 h at 37°C. Sections were rinsed in PBS (3 × 5 min) and incubated for 30 min in a Vectastain ABC kit (Vector Laboratories, Burlingame, California) to amplify the staining, as described by Lecarpentier et al. [47]. The immunoreactivity was visualized using a DAB chromogen with 2% of hydrogen peroxide ammonium nickel for 10 min. Sections were counterstained with toluidine blue and mounted in Eukitt (Sigma Aldrich).

### Stereological analysis

For Cavalieri volume estimation, 3 placentae from early and late gestation and 4 placentae from mid-gestation were used. A series of transversal cross-sections was generated for each placenta. Every 60^th^ section was kept and stained. Stained sections were scanned using a NanoZoomer Digital Pathology System (NDP Scan U10074-01, Hamamatsu, Japan) to produce full panoramic views. The total volumes of the placenta and its compartments i.e., labyrinth, junctional zone, and decidua were determine using the Cavalieri method [2] available on Mercator® software. Each compartment was rigorous outlined manually.

Volume fractions of all the components of the labyrinth area i.e., fetal vessels, sinusoidal giant cells, labyrinthine trophoblast, and maternal blood compartments were quantified by One Stop Stereology using the Mercator® software [48], according to gestational age. For this part, 31 vertical uniform random sections of placenta were used after vimentin immunodetection. Using the software Mercator®, the labyrinth region was outlined manually. Then, around 40–50 probe estimator per section were analyzed as showed in the Figure 1D. The intersection of each categories of cells with the probe estimator was evaluated. In addition, the volume density was automatically calculated by the software.

### Statistical analysis

Statistical analysis was performed using Graphpad Prism®. Kruskall-Wallis was used follow by Dunn’s multiple comparison test. p < 0.05 was considered as statistically significant. All results are expressed as means ± SD.

## Results

Fetal and placental weights of *N. lasiurus* were determined during gestation (Table 1). As expected, their weight increased throughout the gestation by 12.4- and 17.8-fold from early to late gestation, respectively. Moreover the fetal to placental weight ratio, a marker of placental efficiency, increased by 1.19-fold throughout gestation and reached a maximum at 22 days of gestation.

During all gestational stages *N. lasiurus* showed a chorioallantoic placenta organized in three main layers, i.e. the labyrinth, junctional zone and decidua (Figure 1A) like in the *Mus musculus domesticus* (Figure 1B). In the labyrinth, trophoblastic layers (cellular and syncytial), i.e. labyrinthine trophoblast separated maternal and fetal circulations. Whereas, sinusoidal giant cells (one subtype of trophoblast) which showed large size and nuclei were distributed largely in the labyrinth (Figure 1C and D). The junctional zone had a folded structure composed by cellular and syncytial trophoblast and giant cells. In closely contact to the junctional zone, the decidual cells and maternal spiral arteries formed a decidual layer which was attached to the uterus (Figure 1A).

**Figure 2:**
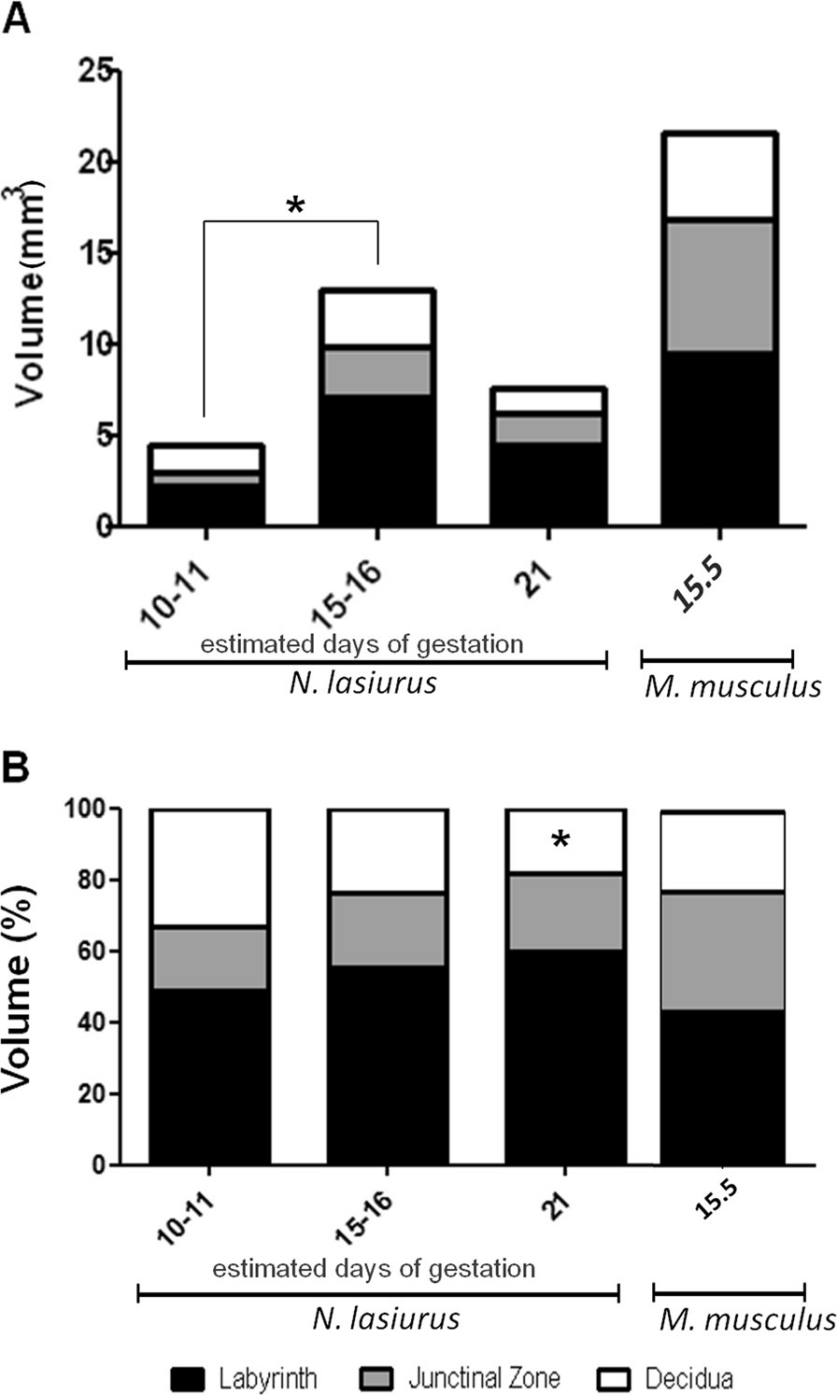
**Volume of *Necromys lasiurus *placenta and of three main layers according to the time of gestation.** (**A**) Total volumes of placenta and of main layers during gestation in *N. lasiurus* and in *Mus musculus domesticus* at 15.5 days of gestation. A significant difference (p < 0.05) was observed in the total volume of the placenta, and in the labyrinth and junctional zone layers between 10/11 and 15/16 days of gestation. There were no statistically significant differences in relation to the volume of the decidua during the gestation. (**B**) Relative volumes of placenta and of main layers during gestation in *N. lasiurus* and *M. musculus* near the term. (*) indicates a significant difference in the volume of decidua between the days 10/11 and 21 days of gestation (p < 0.05). The total volumes of each layer were determined using the Cavalieri method as described in material and methods.

Total and relative volumes of the placentae and of the main layers throughout gestation were estimated by stereology (Figure 2).

### Absolute volumes

A significant increase occurred in the absolute placental volume from early (4.43 ± 0.282 mm^3^) to mid-gestation (12.98 ± 1.305 mm^3^, p < 0.05), then the volume decrease from mid to late-gestation (7.52 ± 0.155 mm^3^) (Figure 2A). Interestingly, the absolute placental volume of *M. musculus domesticus* was 2.87-fold higher than those of *N. lasiurus* at the end of gestation*.* The absolute volumes of the labyrinth and junctional zones and decidua followed the same trend than the absolute placental volume (Figure 2A). In fact, the labyrinth increased significantly its total volume from 2.17 ± 0.078 mm^3^ in early gestation to 7.03 ± 0.945 mm^3^ in mid-gestation (p < 0.05), followed by a decrease to 4.47 ± 0.440 mm^3^ near term (Figure 2A). The total volume of the junctional zone was enlarged from 0.78 ± 0.155 mm^3^ in early pregnancy to 2.82 ± 1.220 mm^3^ in mid gestation (p < 0.05) and decreased to 1.67 ± 0.481 mm^3^ near term (Figure 2A), whereas the total volume of the decidua remained stable throughout the gestation (1.47 ± 0.545 mm^3^ at early stage, 3.122 ± 0.913 mm^3^ at mid-gestation and 1.37 ± 0.270 mm^3^ near term) (Figure 2A). In *M. musculus domesticus* placenta, the absolute volumes of the labyrinth and junctional zone were closed and represented 9.429±1.633 and 7.382±2.142 mm^3^, respectively, in late pregnancy.

### Relative volumes

Relative volume was determined to compare the proportion of each layer at each time of gestation in both *N. lasiurus* and *M. musculus domesticus* placentae (Figure 2B). In *N. lasiurus*, the labyrinth was the most prominent placental layer compared to the junctional zone and to the decidua, especially at the end of gestation when fetal growth is higher. Significant differences (p < 0.05) was found in the relative volume of decidua between 10/11 and 21 days of gestation. In contrast, in *M. musculus domesticus* the proportion of labyrinth layer and junctional zone were similar.

**Figure 3:**
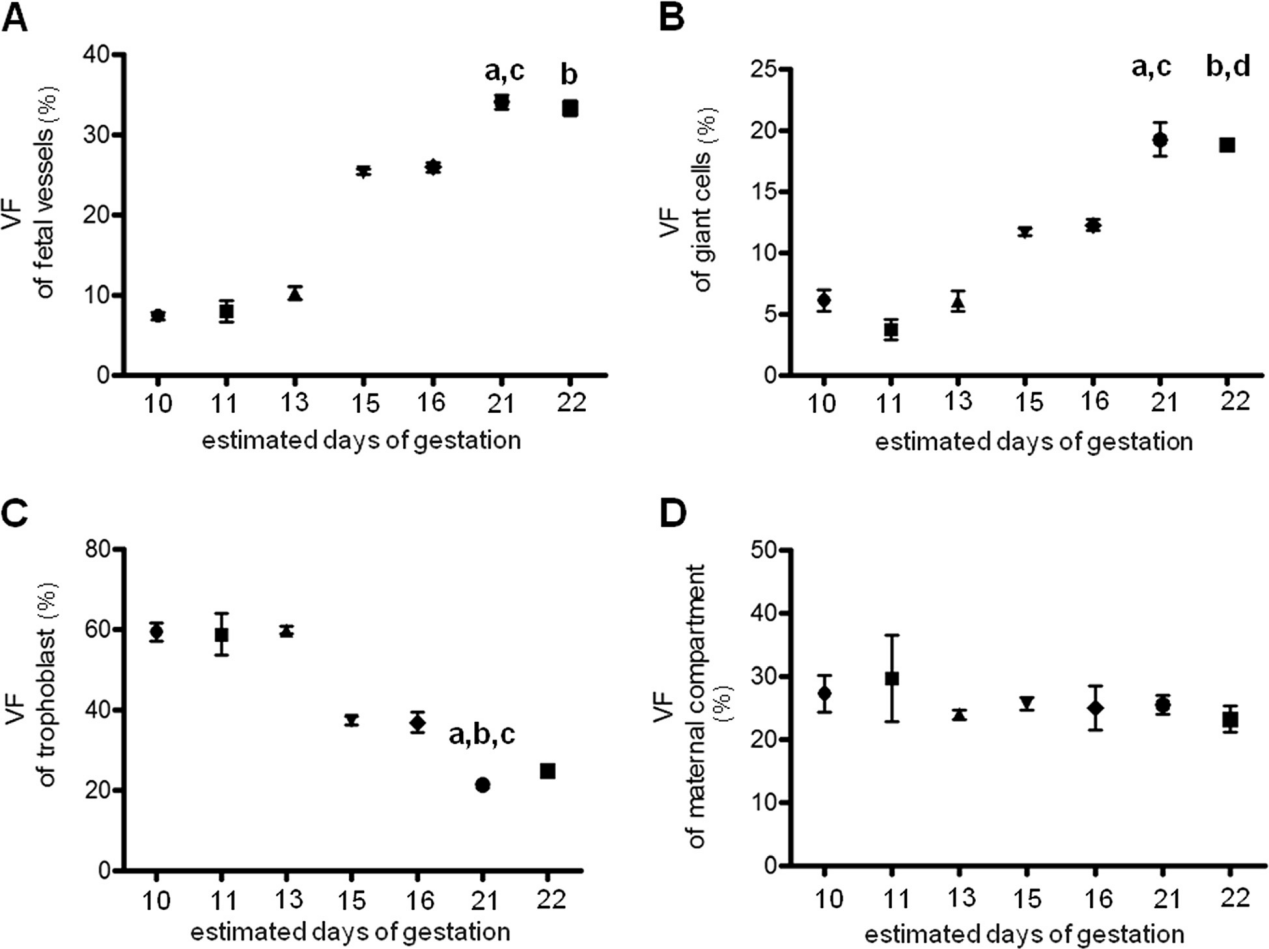
**Determination of volume fraction of labyrinth components during the gestation in *Necromys lasiurus *using One Stop Stereology.** (**A**): Relative volumes fraction of fetal vessels. **a** indicates a significant difference (p < 0.01) between D10 and D21; **b** indicates a significant difference (p < 0.01) between D10 and D22 and **c** indicates a significant difference (p < 0.05) between D11 and D21. (**B**): Relative volumes fraction of sinusoidal giant cells. **a** indicates a significant difference (p < 0.01) between D11 and D21; **b** indicates a significant difference (p < 0.01) between D11 and D22, **c** indicates a significant difference (p < 0.05) between D13 and D21; and **d** indicates a significant difference (p < 0.05) between D13 and D22. (**C**): Relative volumes fraction of labyrinthine trophoblast. **a** indicates a significant difference (p < 0.01) between D10 and D21; **b** indicates a significant difference (p < 0.05) between D11 and D21 and **c** indicates a significant difference (p < 0.01) between D13 and D21. (**D**) There were no significantly differences in relation to the relative volumes fraction of maternal compartment during the gestation. The Mercator® software was used to quantify the volume fractions of all components as illustrated in the Figure 1D.

The volume fractions of the main components in the labyrinth zone during gestation are represented in (Figure 3). A continuous increase in the volume fraction of fetal vessels occurred from day 10 (7.38 ± 0.49%) to day 22 (34.05 ± 0.87%, p < 0.01) (Figure 3A). The volume fraction of sinusoidal giant cells followed the same trend from early pregnancy (6.11 ± 0.84%) to late gestation (19.26 ± 1.34%, p < 0.01) (Figure 3B). In contrast, the volume fraction of the labyrinthine trophoblast was increased in early and mid-gestation (59.29 ± 2.21% and 37.29 ± 1.01% respectively) and decreased significantly at 21 days of gestation (21.16 ± 0.65%, p < 0.01) (Figure 3C). The volume fraction of maternal blood spaces did not undergo significant variation throughout pregnancy (Figure 3D). In *M. musculus domesticus* placentae at day 15.5 of gestation, the labyrinthine trophoblast was the relatively most abundant tissue inside the labyrinth (41.82 ± 0.74%) whereas the volume fraction of fetal vessels, maternal blood spaces and sinusoidal giant cells were lower (23.66 ± 0.44%, 24.48 ± 0.85% and 9.95 ± 0.33%, respectively).

## Discussion

Stereology is a rigorous method, based on mathematical principles and rules for counting, which enables quantitative results in a three-dimensional structure to be obtained using two dimensional samples [2].

Here, we used paraffin embedded placentae for histology and immunohistochemistry. Immunohistochemistry all-owed a better identification of structural components of the labyrinth and consequently lead to a precise analysis of the volumes fractions of *N. lasiurus* and *M. musculus domesticus* placentae.

In contrast to Muridae for which qualitative and quantitative data are available in the literature, only qualitative aspects about the placenta and placentation in a few number of cricetids species were investigated [39-43]. In addition, especially *Necromys lasiurus* have been investigated by our group and detailed qualitative studies about the characteristics of the chorioallantoic and yolk sac placentation were performed using light microscopy, immunohistochemistry, and scanning and transmission electron microscopy [44,49].

In detail, a significant increase in the absolute volume of the placenta and the main regions (labyrinth, junctional zone, and decidua) from early to mid-gestation was observed in *N. lasiurus*, followed by subsequent decrease near term. Thus, maximal placental extension was reached during mid-gestation. This is similar to what was observed in the mouse [9,48,50], supporting the interpretation that general patterns of placental structure and development are largely conserved within the rodent suborder Muroidea. In contrast, the placenta grows continuously in the human, at least under normal conditions, although external factors such as multiple pregnancies and disease could influence this development [5,7,8,51,52]. The differences in growth pattern between the human on the one side and the mouse and its relatives on the other side may have an important influence during gestation. Thus, differential and growth aspects should not be uncritically investigated in mouse model. For instance, Carter [19] provided a balanced and critical discussion on the suitability of the mouse as model species in comparison for human placentation.

It is important to note, however, that the absolute volume of the mouse placenta at the end of gestation ([9,28,50,53], our results) was 3-fold higher than that of *N. lasiurus*, which had a greater body mass. Placental efficiency near term was also more than 10 in the mouse [9,53,54], but only 0.9 for *N. lasiurus*.

The labyrinth was the most prominent placental structure in *N. lasiurus* throughout gestation. In the mouse, at the end of gestation, the absolute volume of the labyrinth was similar to the junctional zone ([9], own results) and its relative size in comparison to the junctional zone was not very different than in *N. lasiurus*. Only in the labyrinth, the fetal capillaries and maternal blood channels are in contact; thus this area has a key role for fetomaternal exchange processes and fetal growth. We can only speculate that the low placental efficiency in sigmodont rodents limits the relative decrease of the labyrinth during pregnancy. The junctional zone contains giant cells, glycogen cells and spongiotrophoblasts (syncytial and cellular trophoblasts) [9,26,44], which may have important endocrine functions in early gestation, but which may not be essential to maintain the exponentially growing fetuses towards term.

Within the labyrinth, the relative proportion of fetal vessels and sinusoidal giant cells increased, whereas that of labyrinthine trophoblast decreased and maternal blood spaces did not exhibit significant changes. These changes may contribute to meet the demands of the developing fetus for adequate nutrition and probably to compensate the low placental efficiency. In that regard, the relative increase in fetal vessels seems to be most important. Moreover, the relative reduction of the labyrinthine trophoblast layer in advanced pregnancy corresponds to a thinning of the three-layered interhemal barrier that optimizes the diffusion distance between the maternal and fetal blood systems [44]. In the mouse near term, the labyrinthine trophoblast becomes the relative most abundant tissue, whereas particularly giant cells are reduced ([9], own results). Optimization for exchange seems to be less significant compared to *N. lasiurus*, which may reflect the effective placentation in the mouse. Finally, in early mouse gestation trophoblastic giant cells maintain important functions related to invasion processes, including the modulation of hormones and growth factor activities [34,55]; their reduction towards term correlate with the establishment of the placenta and the end of invasion. Further studies are necessary to reveal additional functions that may take place especially in the labyrinth by the sinusoidal giant cells in sigmodontine rodents and that may explain the relative explosion in their relative volume during advanced pregnancy, even though trophoblast in general was reduced.

## Conclusion

Though structural aspects of placentation were relatively similar in *N. lasiurus* and the mouse, the quantitative dynamics showed important differences. In particular, the low placental efficiency in *N. lasiurus* seemed to force a more pronounced optimization for fetomaternal exchange processes.

## Competing interests

The authors declare that they have no competing interests.

## Authors’ contributions

POF, MAM, PCP and AT devised the study and participated in its design. POF did the practical analysis, advised by AT. MFO and POF sampled the material. AMM and POF wrote the manuscript. PCP and AT corrected the manuscript. All authors read and approved the final manuscript.
